# Moving epidemic method (MEM) applied to virology data as a novel real time tool to predict peak in seasonal influenza healthcare utilisation. The Scottish experience of the 2017/18 season to date

**DOI:** 10.2807/1560-7917.ES.2018.23.11.18-00079

**Published:** 2018-03-15

**Authors:** Josephine L K Murray, Diogo F P Marques, Ross L Cameron, Alison Potts, Jennifer Bishop, Beatrix von Wissmann, Naoma William, Arlene J Reynolds, Chris Robertson, Jim McMenamin

**Affiliations:** 1 Health Protection Scotland (HPS), Glasgow, United Kingdom; 2 NHS Grampian, Aberdeen, United Kingdom; 3 NHS Greater Glasgow & Clyde, Glasgow, United Kingdom; 4 University of Strathclyde, Glasgow, United Kingdom

**Keywords:** epidemiology, influenza, influenza virus, laboratory, laboratory surveillance, modelling, policy, Scotland, sentinel surveillance, viral infections, statistics

## Abstract

Scotland observed an unusual influenza A(H3N2)-dominated 2017/18 influenza season with healthcare services under significant pressure. We report the application of the moving epidemic method (MEM) to virology data as a tool to predict the influenza peak activity period and peak week of swab positivity in the current season. This novel MEM application has been successful locally and is believed to be of potential use to other countries for healthcare planning and building wider community resilience.

In December of 2017, influenza caused considerable strain on healthcare services. Due to reports that local healthcare systems were under substantial strain over the festive period, in calendar week 52, Health Protection Scotland was asked by the Scottish Chief Medical Officer to predict when influenza activity would peak in primary and secondary care. Such prediction was sought to assist healthcare resilience planning within the context of managing pressures on the National Health Service in the winter [1].

## Problem solving

Methods to predict the time of peak influenza activity were considered. Formal influenza prediction modelling requires additional resource (time, money and statistical expertise) and the validity of output is uncertain. In time-pressured circumstances, this was deemed unfeasible. Use of previous seasons’ laboratory surveillance data to calculate the average number of weeks from start to peak of influenza laboratory detections was contemplated. This option was immediately achievable from existing laboratory surveillance data, required no extra funding and comparatively little extra computational time, and was therefore pursued.

## Data sources

We used two sources of laboratory surveillance data. The first was a primary care virology sentinel swabbing scheme that collates laboratory results of swabs taken from a representative sample of the population who present to primary care with influenza-like illness (ILI) [2]. The second was a secondary care electronic system that collates data from all Scottish laboratories for pathogens including influenza [3]. Over 90% of all samples are submitted from patients presenting to hospital. Positive and negative test results for influenza are received from both schemes allowing the proportion of patients testing positive to be determined, i.e. swab positivity.

## Defining the start of a season

We used the moving epidemic method (MEM) [4] to define the epidemic threshold and identify the start of each influenza season. MEM is a standardised method of reporting influenza activity adopted by the European Centre for Disease Prevention and Control that allows intra- and inter- country comparisons [5]. MEM defines the baseline influenza activity in historical data and establishes an epidemic threshold above which the weekly rates are considered to be in the epidemic period. Based on the historical data, influenza activity intensity is then also described according to categories as follows [6]: (i) baseline: weekly rate ≤ epidemic threshold; (ii) low: epidemic threshold < weekly rate ≤ medium intensity threshold; (iii) medium: medium intensity threshold < weekly rate ≤ high intensity threshold; (iv) high: high intensity threshold < weekly rate ≤ very high intensity threshold; (v) very high: weekly rate > very high intensity threshold.

To calculate the epidemic thresholds for the influenza season 2017/18 we used R [7] and swab positivity data from seasons 2010/11 to 2016/17.

The MEM epidemic threshold for the 2017/18 season (16.7% for primary care virology and 5.7% for secondary care virology) was applied retrospectively to each previous season. The start of a season was defined as the week where swab positivity was and remained above the epidemic threshold for two or more consecutive weeks.

Estimations using the epi-viro proxy parameter (Goldstein indicator) [8] and ILI were conducted, however due to no/infrequent ILI MEM exceedances above baseline, in a number of influenza seasons considered in this work, results from this approach are not presented within the main study findings, but are available for comparison (Supplement). 

## Predicting the peak influenza activity period

For each previous influenza season, using both primary and secondary care virology data, we calculated the week of breach of the epidemic threshold (a); the week of peak in swab positivity (b); the time to peak from breach of threshold in weeks, including the breach week ((b – a) + 1); and the proportion of positives by influenza type/subtype (Tables 1 and 2). We estimated the predicted peak activity period as the average of the time to peak (in weeks) rounded to the nearest whole number, with a range of +/ − 1 week. As influenza A(H3N2) was the predominant subtype of influenza detected this season in Scotland, we estimated the average time to peak using data from the seasons where influenza A(H3N2) was the most prevalent influenza subtype (three seasons for primary and four seasons for secondary care virology).

**Table 1:** Observed primary care virology data^a^ by influenza season in Scotland, 2010/11–2017/18

Season	Week of breach of baseline threshold (> 16.7%) (a)	Week of peak swab positivity (b)	Time to peak in weeks ((b - a) + 1)	Proportion of positives (%)			
				A(H1N1)	A(H3N2)	A (not subtyped)	B
2010/11	48	52	5	51	1	1	47
2011/12	8	11	4	1	84	5	11
2012/13	50	5	8	8	29	2	61
2013/14	5	10	6	84	8	3	5
2014/15	52	5	6	4	67	4	25
2015/16	3	9	7	59	1	2	38
2016/17	50	4	7	< 1	57	10	32
2017/18	47	51	5	2	64	6	29

Seasons with influenza A(H3N2) as the most prevalent subtype are highlighted in light blue for those seasons used to predict peak activity for 2017/18.

^a^ Primary care virology data refer to data from a primary care virology sentinel swabbing scheme that collates laboratory results of swabs taken from a representative sample of the population who present to primary care with influenza-like illness (ILI).

**Table 2:** Observed secondary care virology data^a^ by influenza season in Scotland, 2010/11–2017/18

Season	Week of breach of baseline threshold (> 5.7%) (a)	Week of peak swab positivity (b)	Time to peak in weeks ((b - a) + 1)	Proportion of positives (%)			
				A(H1N1)	A(H3N2)	A (not subtyped)	B
2010/11	50	52	3	68	< 1	3	29
2011/12	8	11	4	1	63	27	9
2012/13	50	52	3	10	49	3	38
2013/14	5	7	3	81	13	3	3
2014/15	51	6	8	2	67	11	20
2015/16	52	10	11	62	2	7	29
2016/17	50	1	4	1	61	13	25
2017/18	47	52	6	2	55	24	19

Seasons with influenza A(H3N2) as the most prevalent subtype are highlighted in light blue for those seasons used to predict peak activity for 2017/18.

^a^ Secondary care virology data refer to data from a secondary care electronic system that collates data from all Scottish laboratories for pathogens including influenza. Over 90% of all samples are submitted from patients presenting to hospital.

Based on primary care virology data, the predicted average time to peak was 6 weeks and the start of the 2017/18 season was in week 47, meaning that the predicted peak activity period would be between week 51 and week 01. Based on secondary care virology data, the predicted average time to peak was 5 weeks and start of the 2017/18 season was also in week 47, meaning that the predicted peak activity period would be between weeks 50 and 52.

**Figure 1:**
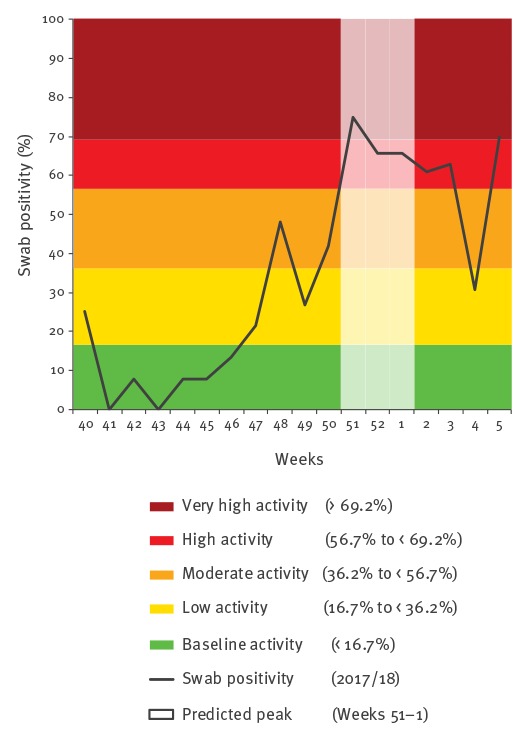
Primary care swab influenza positivity data, moving epidemic method thresholds and predicted peak activity period for the 2017/18 influenza season in Scotland

**Figure 2:**
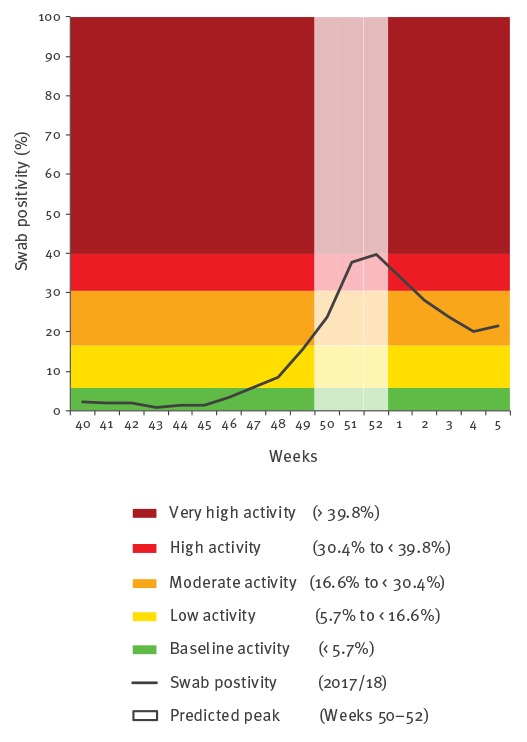
Secondary care swab positivity data, moving epidemic method thresholds and predicted peak activity period for the 2017/18 influenza season in Scotland

These predictions aligned with subsequent swab positivity observations in the 2017/18 influenza season for both primary and secondary care. The observed time to peak in primary care was 5 weeks, and observed time to peak in secondary care was 6 weeks, where peaks were observed in week 51 and week 52 respectively (Tables 1 and 2, Figures 1 and 2).

## Discussion and conclusion

### Application

Understanding the timing of peak seasonal influenza activity is important to Governments, Ministries of Health and resilience planners to inform public health decision making and resource allocation for healthcare system pressures each winter. We propose that the MEM applied to routine virology data may provide a useful tool to define the start of the influenza season and predict the influenza peak activity period.

There are obvious limitations to this predictive approach. First, to our knowledge, no other country has used this prediction of peak activity before, so we have no means to benchmark our work. Second, we have not predicted the severity of the season to come, however we look forward to output from World Health Organization Pandemic Influenza Severity Assessment (PISA) project [9] and others on this matter.

Third, we were unable to calculate retrospective season-specific MEM thresholds due to lack of available data before season 2010/11. The retrospective application of 2017/18 thresholds was used as a pragmatic approach and believed to be accurate due to the small variation between epidemic thresholds in recent seasons (Supplement).

Fourth, laboratory data are affected by reporting delay, especially during festive periods, which may affect the ability to timely detect the start of the season. Repeated predictions would account for this when more data become available.

We are mindful that our vaccine uptake, the effectiveness of the vaccine and the public health impact of the influenza vaccination programme will play a role in mitigating our current influenza season [10,11], however, the precise impact is unknown. It is important to consider this when further validating the usefulness of the above method.

The strength of this approach is its relative low time and data requirements, which allow a quick and simple estimation of the likely peak influenza activity period. This was possible due to the fact that Scotland has a well-established, comprehensive, representative and timely influenza surveillance system with enough retrospective electronic data available to allow application of the MEM methodology.

In this study, while applying the Goldstein indicator was considered (Supplement), there were two influenza seasons (2011/12 and 2013/14) where this was not possible because the data only breached the epidemic threshold in one and two weeks, respectively. There was moreover a similar limitation for ILI – in both the influenza seasons 2011/12, 2013/14, ILI did not breach the MEM threshold. Indeed, since the successful introduction of the live adapted intranasal influenza vaccine (LAIV) in children (LAIV vaccine uptake ≥50% in pre-school children and ≥70% in primary school age children) in Scotland [12] we have observed no/infrequent ILI MEM exceedances above baseline (2014/15, 2015/16, 2016/17). A pragmatic approach was therefore to use the best available data, consisting of swab positivity data from our general practice sentinel swabbing scheme. 

Due to the pragmatic nature of our methods, our prediction was used in real time, and at short notice to inform resource planning to ensure the population healthcare system continued to function amid challenges posed by influenza. Furthermore, as this approach did not require significant additional resource, we believe this low-cost method could be applied widely.

This MEM approach made two key assumptions. Firstly, that the pattern from the past would be indicative of the pattern that we would see in the current season. Secondly, that timing would be the same in all influenza seasons where influenza A(H3N2) was the most prevalent subtype.

We also tested the hypothesis that the timing would be the same regardless of influenza subtype by using all influenza seasons data (data not shown). This did not change the weeks predicted, therefore we cannot be certain whether the most prevalent subtype of influenza plays any role in prediction. This may suggest that our approach could be useful for planning regardless of the seasonal dominant influenza subtype.

### Next steps

Further work examining the usefulness of MEM for seasons dominated by other influenza types or the influence of co-dominance of influenza type/subtype needs to be undertaken. Similarly, the application of season-specific MEM thresholds should be attempted in the future.

We have written this rapid communication to allow colleagues in other countries to explore the usefulness of applying the same methodology to predict peak influenza activity in their countries. This may allow them to inform resilience planning and policy for healthcare systems to respond effectively to winter pressures. We look forward to their findings.
